# A routine check‐up at the digital medical centre

**DOI:** 10.1111/1751-7915.12454

**Published:** 2016-12-01

**Authors:** Kenneth N. Timmis, James K. Timmis

**Affiliations:** ^1^ Department of MicrobiologyTechnical University of BraunschweigBraunschweigGermany; ^2^ Albert‐Ludwigs‐Universitát FreiburgFreiburg im BreisgauGermany

*(Mrs. Tristram‐Wellness has just received an automatic e‐notification that her routine health check‐up is due, and arrives at the entrance of the Digital Medical Centre. She proceeds to the patient identification booth, lays her thumb on the fingerprint scanner, and stares into the iris scanner. After identification confirmation, she enters the adjoining basic health parameter room and follows the on‐screen instructions for measurement of pulse and blood pressure, and weight and height, and then responds to the questions posed about nutrition, lifestyle and other health‐related issues. After the pre‐consultation assessment, she exits to the corridor, activates the facultative body‐craniofacial scanners, and walks purposefully, head up, along the corridor to a private booth for her consultation.)*

Dr. Siri von App^1^: Good morning Mrs. Tristram‐Wellness. Thank you for coming for your scheduled check‐up; how are you today?

Mrs. Tristram‐Wellness: Hello Doctor, I am very well, thank you.

Dr. SVA: Good. So, firstly, the scanners did not pick up anything unusual in either your gait, diagnostic craniofacial characteristics, or breathing‐temperature features, and the pre‐consultation assessment parameters are perfectly normal.

Now, I see from your records that to control your multiple sclerosis condition you had a nanoimmunomonitoring‐dosing device^2^ implanted 18 months ago. According to the multiparametric data it automatically uploads to your medical profile in our database, it seems to be managing your condition effectively. Do you tolerate the implant well and does it mitigate your MS symptoms satisfactorily?

Mrs. TW: Yes, I hardly notice the implant and it has really improved my quality of life.

Dr. SVA: Excellent! Last time you came for a check‐up, you complained of tiredness in the afternoon: have the oral genome‐matched intestinal microbial cocktail and prebiotic capsules, coupled with the lunchtime energy drink I prescribed, helped at all?

Mrs. TW: Yes, definitely: in fact they improved things so much so that I was able to join an afternoon Zumba class twice per week. However, I now feel tired in the evenings.

Dr. SVA: Do you regularly drink alcohol in the evening?

Mrs. TW: Well, yes: we drink a modest amount during supper – no more than 1–2 glasses – of a varietal wine from Chile, made from a Carmenere grape engineered in California to produce elevated levels of resveratrol and procyanidins, and fermented with a minimal genome synbiol yeast which, I have learned, was designed by a joint Spanish‐Chinese consortium to produce no acetic acid and several undisclosed compounds from plants used in traditional Chinese medicine.

Dr. SVA: Impressive, but it's still alcohol, right? Your tiredness probably results in part from the vigorous exercise, and in part from the evening alcohol consumption, and, according to my analysis of the available evidence, is probably not due to an underlying health issue. I suggest that you reduce your consumption of alcohol to minimize alcohol‐induced tiredness.

Your records show that, at the time of your last check‐up, you were pregnant and that the birth (congratulations, by the way) was without incident.

Mrs. TW: Oh, yes: the initial discovery and subsequent genetic correction of the paternally‐inherited diabetes predisposition and *in vitro* fertilisation procedure were very reassuring from the outset, and my current partner and I are over the moon with the lovely addition to our family.

Dr. SVA: Excellent! And did you decide to take the opportunity to have your baby's genome sequenced at birth, and to commit to regular microbiome assessments?

Mrs. TW: Most certainly, Doctor! We believe that these measures are vital to the optimisation of lifestyle choices for good health and disease prevention and treatment. And, of course, we are pleased to be able to support medical research, albeit in a minor way, through contributing our anonymized genome and microbiome sequences, and medical histories, to the national genome database.

In fact, my 9‐year‐old son, George, interrogates the family genome sequences every time a new algorithm becomes available, and knows all kinds of things about our susceptibilities and strengths. It is amazing what topics amuse children these days! For example, he discovered his predisposition towards diabetes and understood the heightened risk resulting from obesity exacerbated by spending too much time playing traditional (online) computer games and not getting enough exercise. As a result, we were able to install on our virtual reality system some new generation movement‐centred gaming programmes. These have in the meantime become highly popular family pastimes, much to the dismay of my partner, who frequently has to reschedule his virtual ocean fishing trips with his friends who live across the globe.

And George cheerfully announced over breakfast this morning that my partner, who, it has to be admitted, is a rather orderly individual, has a genetic predisposition to a mild form of progressive obsessive–compulsive personality disorder. Fortunately, it appears that there are now third‐generation SSRIs in the pipeline with fewer side‐effects than current drugs that should be able to control the condition as it progresses. Wickedly, George then proceeded to *checkmate* by exclaiming that complaints about the disorderly state of his bedroom were unjustified and provoked merely by my partner's condition!

Dr. SVA: Hhmm….. I see. Moving down my checklist…..as you know, domestic animals are important for establishing in infants a healthy immune system associated with a low incidence of allergies in later life, and maintaining healthy microbiomes in family members^3^. Last time you were here, you indicated that you did not have a pet: is this still the case?

Mrs. TW: No: we now have a wonderful second‐generation child‐optimized spaniel‐retriever cross which sleeps at the bottom of our baby's bed at night. He is so gentle with all children – ours and their friends – some of whom are, I fear, rather rough and not infrequently cruel to him. The genetic elimination of the hunting‐fighting trait in this breed has been a wonderful development, though it is pity about the brittle bone syndrome and social dysfunctionality vis‐a‐vis other dogs!

Dr. SVA: True, but an excellent choice nevertheless!

Now, please go to the nurse in the clinical‐environmental parameters booth who will take a blood sample for comprehensive determination of metabolic‐immunological‐endocrinological parameters, and skin and throat swabs for microbiome profiling. She will also provide you with containers for faecal and urine samples, which you should return to her within 2 h of taking the samples, and a device for collecting and analysing the air contents of the rooms of your home and place of work^4^. Please complete the room sampling within 7 days and promptly return the *Airalysis* module for re‐commission. The analyses it carries out will be automatically uploaded into your medical profile in the national database, and will determine whether or not we will recommend you to use an ectoine inhaler, given that your place of work is on a brownfield site recently shown to be emitting low levels of VOCs (*Au: volatile organic compounds*).

In addition, the nurse will assess your immunization profile and upcoming travel plans, and propose a personalized series of new vaccinations. As you will have heard, healthcare is becoming increasingly prevention‐centric and, in addition to the availability of new generation vaccines for malaria, Zikavirus, water‐food‐borne strains of hepatitis, etc., a number of other vaccines have been introduced as a result of recent studies on the broad benefits of vaccination^5^, ^6^ and the harmonization of universal priorities for vaccine evaluation and introduction.^7^

After all results have been received and clinically evaluated, you will automatically receive an MFGCBEL (*Au: multi‐factorial genomic‐centric, biome‐environment‐lifestyle*) report which will also inform you if it is necessary to come in again to discuss any appropriate recommendations for changes in your health regime.

Do you have any other issues you would like to discuss?

Mrs. TW: Well, yes: I do not intend to have any more children, so am now beginning to think about my post‐parental period and what unfulfilled achievement potential my genome might indicate. I should therefore appreciate your running my genome through the physiological‐intellectual‐talent scoring programme, and inform me of further development potential I may have and which training would take advantage of this potential to elicit fulfilment of my abilities.

Dr. SVA: Ok……this will take a few moments……..right: I see that you have excellent lung function and, concerning your genome, it seems that you may have significant potential for further increasing both your air volume intake and gas exchange function, which would enable you to develop excellent underwater diving capabilities. This would be one option for potential fulfilment, which also has the advantage that it could involve travel to beautiful marine locations. I will have a personalised lung function development and diving training programme sent to your secure private access patient interface.

Do you have any other questions?

Mrs. TW: Yes: I recently read on the internet (*Dr. SVA: audible sigh*) that the eggs of the greater spotted quivering bottom butterfly contain a substance that prevents skin sag during ageing, but the product is eye‐wateringly expensive. Nevertheless, I wondered if you would recommend it?

Dr. SVA: Well, since the gsqbbutterfly is on the endangered species list, it is illegal to acquire its eggs, so any that become available are of questionable provenance. For this reason, there also exists no clinical evidence of the substance's efficacy and effectiveness in preventing skin sagging. However, the closely‐related lesser spotted quivering bottom butterfly is an abundant pest and has been studied extensively. The only pharmacologically active substance thus far found in its eggs has been isolated, characterized and chemically synthesized. Bioinformatic‐modelling analyses of the potential activities of all possible chemical decoration variants of the scaffold of the substance, combined with human systems biology studies, do not suggest any significant beneficial activity relating to skin tonality, so I cannot recommend your pursuing this idea further.

Mrs. TW: (*sigh*) Oh, okay then.

Dr. SVA: One final point: I am not entirely certain that the perfume you are wearing really suits you. I might suggest that you make an appointment with the Biome Odour‐Olfactory Clinic for a perfume:personal skin microbiome compatibility‐optimisation analysis^8^, and explore some of the personalised perfumes they will be able to propose.

Mrs. TW: Oh……well.. I must say this comes as a bit of a surprise, Doctor, but I will follow your advice! Thank you.

Dr. SVA: You are welcome. Goodbye.

## Notes

1 Au: Dr. SVA is a third‐generation, humanized smart database interrogation device (HSDID)

2 Guzman, C.A. and Timmis, K.N. (2011) Towards intelligent vaccines: the VAC‐CHIP, in Crystal ball‐2011. *Microb Biotechnol* **4:** 109‐137.

3 Fujimura, K.E. and Lynch, S.V. (2015) Microbiota in allergy and asthma and the emerging relationship with the gut microbiome. *Cell Host Microbe* **17**: 592‐602.

4 Gordon, J., Gandhi, P., Shekhawat, G., Frazier, A., Hampton‐Marcell, J., Gilbert, J.A. (2015) A simple novel device for air sampling by electrokinetic capture. *Microbiome* **3**: 79.

5 Barnighausen, T., Bloom, D.E., Cafiero‐Fonseca, E.T., O'Brien, J.C. (2014) Valuing vaccination. *Proc Natl Acad Sci USA* **111**:12313‐9.

6 Bloom, D.E. (2015) Valuing vaccines: deficiencies and remedies. *Vaccine* **33**(Suppl. 2): B29‐B33.

7 Timmis, J.K., Rigat, F., Rappuoli, R. (2017) Core values for vaccine evaluation. *Vaccine* (in press).

8 Timmis, K. (2009) Human biome biotechnology and the personalization of odour profiles. *Microb Biotechnol* **2**: 150‐152.

